# Signatures of human European Palaeolithic expansion shown by resequencing of non-recombining X-chromosome segments

**DOI:** 10.1038/ejhg.2016.207

**Published:** 2017-01-25

**Authors:** Pierpaolo Maisano Delser, Rita Neumann, Stéphane Ballereau, Pille Hallast, Chiara Batini, Daniel Zadik, Mark A Jobling

**Affiliations:** 1 Department of Genetics, University of Leicester, Leicester, UK

## Abstract

Human genetic diversity in Europe has been extensively studied using uniparentally inherited sequences (mitochondrial DNA (mtDNA) and the Y chromosome), which reveal very different patterns indicating sex-specific demographic histories. The X chromosome, haploid in males and inherited twice as often from mothers as from fathers, could provide insights into past female behaviours, but has not been extensively investigated. Here, we use HapMap single-nucleotide polymorphism data to identify genome-wide segments of the X chromosome in which recombination is historically absent and mutations are likely to be the only source of genetic variation, referring to these as phylogeographically informative haplotypes on autosomes and X chromosome (PHAXs). Three such sequences on the X chromosome spanning a total of ~49 kb were resequenced in 240 males from Europe, the Middle East and Africa at an average coverage of 181 ×. These PHAXs were confirmed to be essentially non-recombining across European samples. All three loci show highly homogeneous patterns across Europe and are highly differentiated from the African sample. Star-like structures of European-specific haplotypes in median-joining networks indicate past population expansions. Bayesian skyline plots and time-to-most-recent-common-ancestor estimates suggest expansions pre-dating the Neolithic transition, a finding that is more compatible with data on mtDNA than the Y chromosome, and with the female bias of X-chromosomal inheritance. This study demonstrates the potential of the use of X-chromosomal haplotype blocks, and the utility of the accurate ascertainment of rare variants for inferring human demographic history.

## Introduction

Studies of the origins and histories of European human populations have been transformed by the availability of next-generation sequencing (NGS), which has given access to the genomes of ancient humans and allowed the unbiased ascertainment of sequence variants in modern populations. Autosomal sequence data from ancient remains have demonstrated discontinuity between Palaeolithic hunter-gatherers and Neolithic farmers,^1, 2, 3, 4^ and more recently have pointed to a later shift because of mass migration from the Pontic-Caspian steppe during the Bronze Age.^5, 6, 7, 8^ In modern populations, NGS-based studies of uniparentally inherited loci (the male-specific region of the Y chromosome (MSY) and mitochondrial DNA (mtDNA))^9, 10^ have also suggested the importance of recent changes, with marked differences between the two systems being attributed to male-specific Bronze Age expansion.^11^ By contrast, information emerging from resequencing the autosomal genomes of modern individuals has been of limited utility in understanding the events of European prehistory.

The properties allowing MSY and mtDNA to provide useful insights into the past are their haploidy and lack of recombination, which permit demographic reconstruction from haplotypes, and their uniparental inheritance, which provides a sex-specific aspect to the demographic inferences. In this light, the X chromosome represents a potentially useful additional source of information^12^ that has yet to be fully exploited. It is inherited twice as frequently from mothers as from fathers and therefore contains a record biased towards past female behaviours. In males, it is haploid, so sequencing in males provides unambiguous phasing of haplotypes, including those bearing rare variants. Finally, it shows high average levels of linkage disequilibrium (LD) because most of its length is exempt from crossover in male meiosis. It should therefore be possible to identify segments of the X chromosome that have histories of little or no recombination, to determine their sequences unambiguously in modern male samples using NGS methods and to use demographic reconstruction to infer female-biased histories. Moreover, recent work has focused on the X chromosome to derive sex-biased admixture models with constant ongoing admixture,^13^ and segments of the X chromosome showing little or no recombination would be suitable markers for these approaches as well.

A number of resequencing studies of X-chromosomal loci have been carried out previously. These have generally surveyed segments of 1–10 kb in global samples,^14, 15, 16, 17, 18, 19, 20, 21, 22^ and have demonstrated excess gene diversity in African compared with non-African populations, consistent with observations from the autosomes.^23^ Some of these studies have chosen segments in which recombination is expected to be low,^19, 20, 21^ allowing the relatively simple construction of gene trees, while others have not used low recombination rate as a selection criterion, and have yielded haplotypes presenting evidence for multiple recombination events.^17^ To the authors' knowledge, a population-based study of X-chromosomal sequence diversity in Europe has not yet been undertaken.

Here, we select three segments of the X chromosome that show no historical recombination in HapMap data, and use a population resequencing approach to analyse these in multiple European population samples, as well as single Middle Eastern and African population samples. Our findings show that the non-recombining nature of these segments persist, the histories of these X haplotypes in Europe are dominated by Palaeolithic expansions and suggest that larger-scale investigation of the X chromosome will provide further useful insights into the European past.

## Materials and methods

### Samples

Two hundred forty DNA samples were analysed, comprising 20 randomly chosen males from each of 12 populations. The list of samples including population origins is reported in Supplementary Table S1 and additional details were previously described.^11^ An additional 13 unrelated male samples (see Supplementary Material) from six populations were analysed from the Complete Genomics sequence data set.^24^

### PHAX identification process

Phylogeographically informative haplotypes on autosomes and X chromosome (PHAXs) were originally defined using publicly available single-nucleotide polymorphism (SNP) data from the HapMap project,^25^ release 21, for four population samples: the Centre d'Etude du Polymorphisme Humain (CEPH) collection in Utah, USA, with ancestry from Northern and Western Europe (CEU), the Yoruba from Ibadan, Nigeria (YRI), Han Chinese in Beijing, China (CHB) and Japanese in Tokyo, Japan (JPT). Haplotypes were inferred using PHASE^26^ and measures of LD were downloaded from the HapMap website, release 16c.

Historically non-recombining regions were identified on both autosomes and X chromosome as non-overlapping series of at least three adjacent SNPs where each pair had a |D'| value of 1 in each of the three samples CEU, YRI and JPT+CHB, and for which only three of the four possible two-allele haplotypes were observed in the entire sample set, including the ancestral haplotypes (inferred from chimpanzee data), whether or not these were themselves observed. See Supplementary Material for additional details.

Candidate PHAXs for resequencing were chosen to be: (i) free of genes; (ii) separated from the nearest known or predicted gene by at least one recombination hotspot; (iii) lacking in segmental duplications, for ease of sequence interpretation; and (iv) possessing an ortholog in the chimpanzee genome,^27^ for convenience of ancestral state determination. PHAXs passing these filters were sorted by the number of haplotypes defined by SNPs in the CEU sample,^25^ given that the focus of our study was on European populations. We recognise that choice on the basis of diverse haplotypes may represent a source of bias, and will address this in a future study by analysing a larger random set.

### Amplicon sequencing, data analysis, variant calling and filtering

The top three X-chromosomal PHAXs were divided into 10 ~5-kb amplicons (including short overlaps) for resequencing. In all, ~10 ng of genomic DNA was used for amplification of the three chosen PHAXs via each of 10 amplicons, through polymerase chain reaction (PCR). Amplicons were quantified on 0.8% (w/v) agarose gels, and pooled at equimolar concentrations per sample. For each sample, 100 ng of amplified pooled DNA was used for library preparation, via the Ion Xpress Plus gDNA Fragment Library Preparation kit (Thermo Fisher Scientific, Loughborough, UK). Size selection was done using Agencourt AMPure XP beads (Beckman Coulter, High Wycombe, UK). Ion Xpress barcodes were used to tag individual libraries, which were quantified using the 2100 Bioanalyzer (Agilent Technologies, Santa Clara, CA, USA) and the Agilent High Sensitivity DNA Kit. An equimolar pool of libraries was fractionated on a 2% (w/v) NuSieve 3:1 agarose gel. Final size selection and clean-up were performed with the Zymoclean Gel DNA Recovery Kit (Zymo Research, Irvine, CA, USA). Template preparation was carried out using the Ion Xpress Template 200 Kit (Thermo Fisher Scientific), before sequencing on an Ion Torrent PGM platform with 200-bp reads using the Ion PGM Sequencing 200 Kit (Thermo Fisher Scientific) and the Ion 316 chip v1.

Reads were mapped to the human reference sequence (hg19) using TMAP software implemented in the Ion Alignment plugin 3.2.1 (Torrent Suite Software 3.2.1, Thermo Fisher Scientific). Local realignment and duplicate read marking were carried out with the Genome Analysis Tool Kit (GATK)^28^ and picard v1.94 (http://picard.sourceforge.net/), respectively. All sites were called using SAMtools 0.1.19^29^ and filtering was done with in-house scripts. A total of 49 070 bp were called, including 419 raw variants from 240 samples. Following filtering, 297 variants and 238 samples were retained.

*In silico* validation was done using Complete Genomics whole-genome sequence data (http://www.completegenomics.com/public-data/69-genomes/; nine samples) and Illumina sequence-capture data^30^ using only shared called sites in the PHAXs sequenced here (220 samples). Based on the complete genomics comparison, the false-positive rate was 0.0005% and false-negative rate 0, whereas via the Illumina comparison the false-positive rate was 0 and the false-negative rate 0.00003%. Further details on filtering and data analysis are reported in the Supplementary Material.

### Intra- and inter-population diversity

Haplotype diversity,^31^ Tajima's D^32^ and Fu's Fs^33^ were calculated for each PHAX per population using Arlequin v3.5.^34^ Genetic differentiation between populations was measured with the molecular index ϕst,^35^ computed with Arlequin v3.5.^34^

### Networks and BSPs

Relationships between different haplotypes were displayed in median-joining networks,^36^ implemented in Network 4.6 (http://www.fluxus-engineering.com/sharenet.htm). Ancestral state for each site was defined by comparison with the chimpanzee reference sequence.

Bayesian Skyline Plot (BSP) analyses were performed using BEAST v 1.8.0.^37^ Markov chain Monte Carlo samples were based on 200 000 000 generations, logging every 10 000 steps, with the first 20 000 000 generations discarded as burn-in. Traces were evaluated using Tracer v 1.6 (http://beast.bio.ed.ac.uk/software/tracer/). A piecewise linear skyline model with 10 groups was used with a Hasegawa, Kishino and Yano substitution model^38^ and a strict clock with a mean substitution rate of 6.59 × 10^−10^ mutations/nucleotide/year (details in Supplementary Material). A generation time of 30.8 years was used.^39^

### TMRCA estimation

TMRCA estimation for specific haplotype clusters was performed using the rho statistic,^40, 41^ using Network 4.6 (http://www.fluxusengineering.com/sharenet.htm). This analysis was performed on individual PHAXs with a scaled mutation rate of 41 171, 304 525 and 263 309 years per mutation for PHAX 5574, 3115 and 8913, respectively. These estimates were based on the mutation rate (6.59 × 10^−10^ mutations/nucleotide/year) and the number of nucleotides for each PHAX (36 857, 4983 and 5763 for PHAX 5574, 3115 and 8913, respectively).

## Results

### Selection of non-recombining X-chromosomal regions

We used genome-wide HapMap data and LD analysis (Supplementary Material) to identify regions showing no evidence of historical recombination in 210 unrelated individuals from the four populations CEU (Utah Residents with Northern and Western European ancestry), YRI (Yoruba in Ibadan, Nigeria), JPT (Japanese in Tokyo, Japan) and CHB (Han Chinese in Beijing, China). Regions passing our LD filters were designated PHAXs.

**Figure 1:**
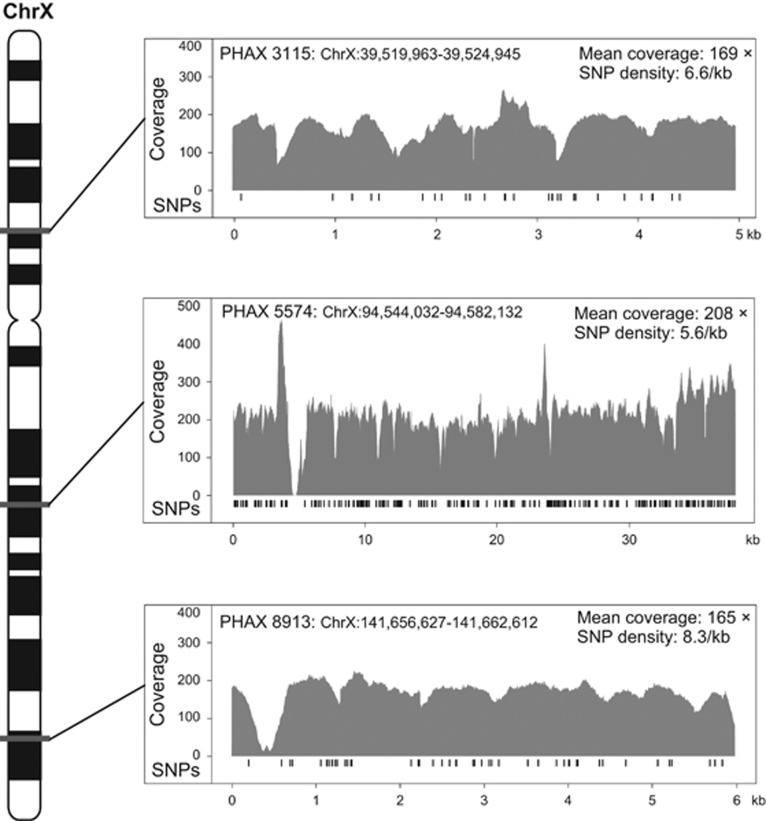
Location, sequence coverage, and SNP distributions for the three PHAXs studied. The approximate positions of the three studied PHAXs are shown on the X-chromosomal ideogram to the left. To the right the three panels show the average coverage of sequence reads across samples per site and the position of SNPs (black bars) in each PHAX. Mean coverage and SNP density are also shown.

For the purposes of this study, we applied additional filters to the set of PHAXs identified on the X chromosome in order to obtain a set of informative, independent and putatively neutrally evolving markers. The top three regions were selected, spanning ~49 kb in total (Table 1, Figure 1, Supplementary Material).

### Genetic diversity and data summary

**Figure 2:**
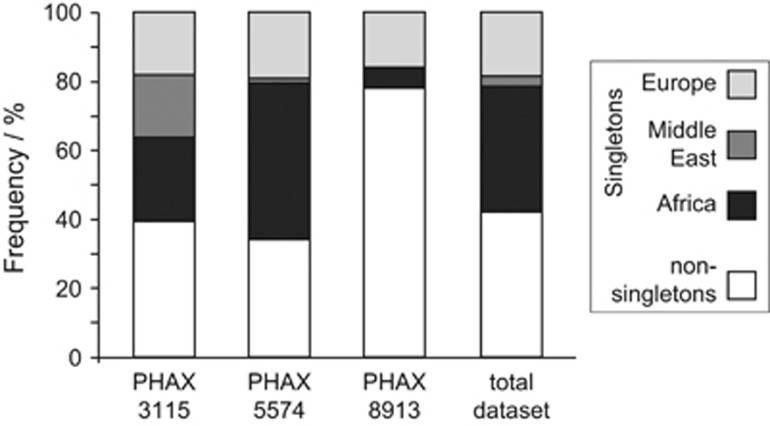
Distribution of singleton and non-singleton variants. Histogram showing distribution of variant types by PHAX, and by meta-population. Africa is represented by YRI (Yoruba from Ibadan, Nigeria); Middle East by Palestinians; Europe by the remaining 10 populations.

We used Ion Torrent sequencing to assess the genetic diversity of the three X-chromosomal PHAXs in a total of 240 men from Europe, the Middle East and Africa (Supplementary Table S1). Mean coverage was 181 × and we called all sites (ignoring indels) having ≥10 × coverage (Figure 1). SNPs were validated *in silico* by comparison with published whole-genome sequences (http://www.completegenomics.com/public-data/69-genomes/), and with a subset of the same samples and target regions sequenced using Illumina technology.^30^ The high coverage and high threshold for variant calling led, respectively, to very low false-negative and false-positive rates (Supplementary Material). We ascertained 297 high-quality SNPs in total, which defined 29, 78 and 30 distinct haplotypes, respectively, for PHAX 3315, 5574 and 8913 (Supplementary Tables S2-S4). Fifty-eight of the SNPs (19.5%) were not previously reported in dbSNP build 138, and over half (172; 57.9%) were singletons (Figure 2), that is, unique in the data set. PHAXs 3115 and 5574 were significantly enriched in singletons (Fu and Li's D test –5.48 and –7.88, respectively, with both *P*-values <0.02). SNP density varies among the three PHAXs and the difference is marginally significant (chi-square test with Yates' correction, chi-square=6.024, *P*=0.045) suggesting different evolutionary histories for these loci. This was also supported by the significant pairwise differences between the distributions of haplotype diversity of the three PHAXs (Kolmogorov–Smirnov test *P*-values 0.000084, 0.03 and 0.03 for the comparisons PHAX 5574 *vs* PHAX 8913, PHAX 5574 *vs* PHAX 3115 and PHAX 3115 *vs* PHAX 8913, respectively).

### Descriptive analyses

Population structure was investigated by performing a principal components analysis (PCA) based on haplotype frequencies within populations. Overall, the first two PCs combined together explain a total of 32% and 61% of variance for the three PHAXs. When all populations were analysed together, the YRI sample was consistently separated from non-African populations in all three cases (Supplementary Figure S1). To better examine the population structure of non-African populations (Supplementary Figure S2), the YRI sample was removed in each case, whereas the Palestinian population was omitted for PHAX 3115 only, as it was an outlier (Supplementary Figure S2a). All plots show a lack of specific clustering and structure. This pattern was confirmed by the pairwise ϕst matrix (Supplementary Table S5), which shows strong differentiation between the YRI and other samples, but similar genetic distances among all non-African populations. Overall, genetic distances do not suggest significant population structure within Europe.

### Phylogeographic analysis

**Figure 3:**
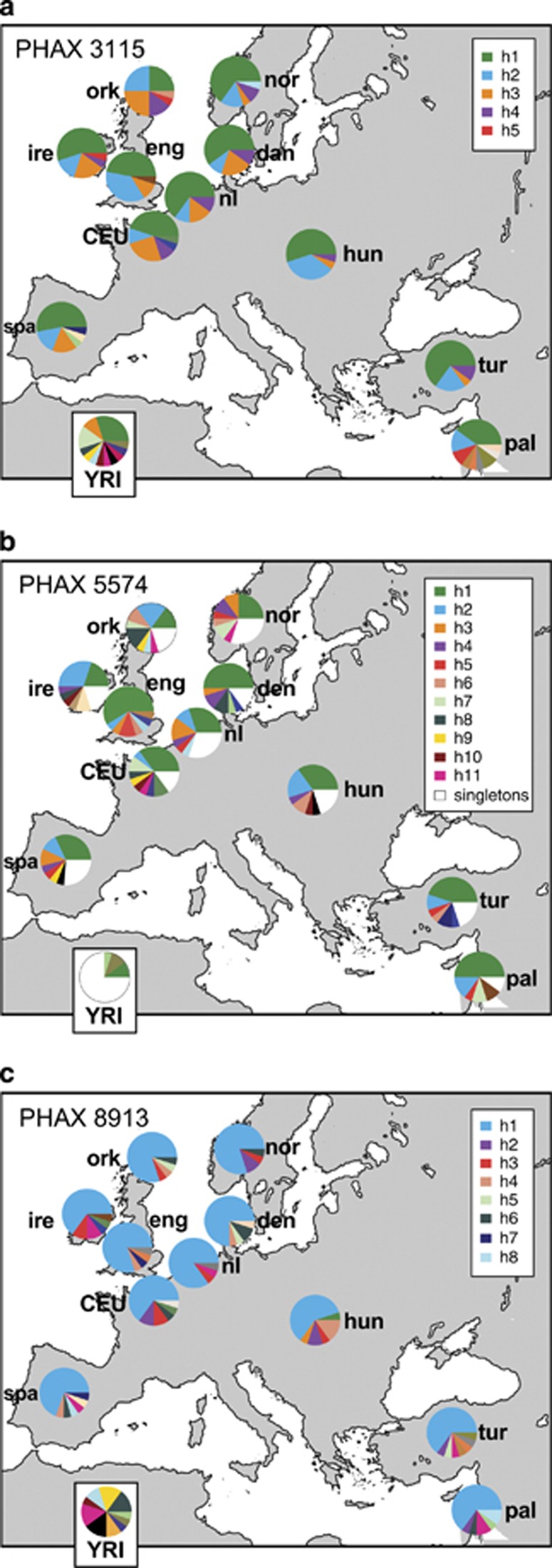
Population distributions of haplotypes. Maps showing distributions of haplotypes for each PHAX, indicated by coloured sectors in pie charts. At the bottom of each panel, the distribution for the YRI population is also shown. (**a**) PHAX 3115: the key indicates non-singleton non-African haplotypes (h1–5). (**b**) PHAX 5574: the key indicates haplotypes (h1–11) present in three or more non-African individuals; white sectors in pie charts correspond to all singleton haplotypes, which are numerous for this PHAX. (**c**) PHAX 8913: the key indicates non-singleton non-African haplotypes (h1–8). Population abbreviations are as follows: CEU: Utah residents with Northern and Western European ancestry from the CEPH collection (French); den, Danish; eng, English; nl, Dutch; hun, Hungarian; ire, Irish; nor, Norwegian; ork, Orcadian; pal, Palestinians; spa, Spanish; tur, Turkish; YRI, Yoruba from Ibadan, Nigeria.

Haplotype frequencies were plotted per population according to their geographic locations (Figure 3). For all three PHAXs, the YRI sample shows a high number of haplotypes at low and intermediate frequencies (up to a maximum of 18 haplotypes from 20 individuals for PHAX 5574). In Europe, populations carry a few haplotypes at high frequencies and many at low frequencies (0.05). Some local geographical patterns in haplotype distributions can also be observed. For example, haplotype h2 of PHAX 3115 (Figure 3a) is not present among the YRI but is relatively frequent in Europe, whereas haplotype h3 (Figure 3a) is at relatively high frequency in Central and Western Europe, but also persists at low frequency in Middle East and the YRI. Such patterns are more pronounced for PHAX 5574 and PHAX 8913. Haplotype h2 of PHAX 5574 (Figure 3b) is present only in Europe and the Middle East with its highest frequency in Ireland. PHAX 8913 shows a common haplotype (h1) with frequency ranging from 0.6 to 0.8 in Europe that is absent from the YRI (Figure 3c) – features that could indicate a founder effect associated with European colonisation.

**Figure 4:**
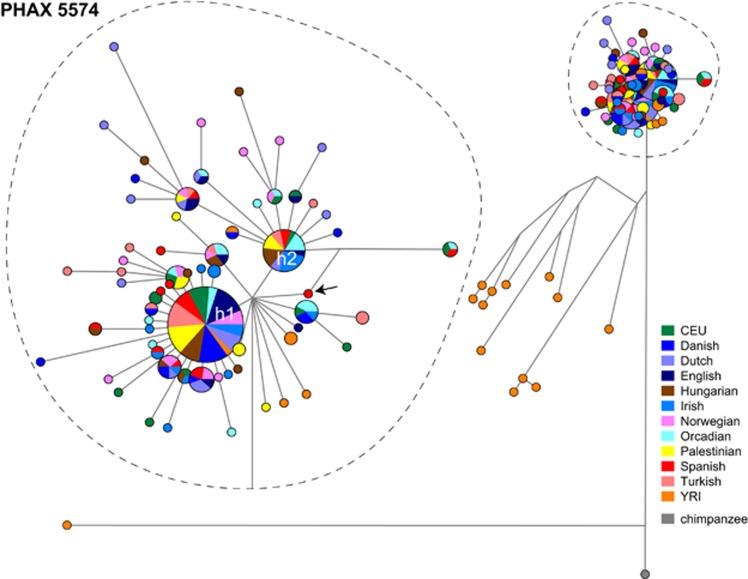
Median-joining network for PHAX 5574 showing population distribution of haplotypes. Circles represent haplotypes with area proportional to frequency, and lines between them represent SNP mutational steps between haplotypes. Populations are indicated by colours as shown in the key to the right. The major haplotype cluster is magnified in the dotted ellipse to the left for clarity, and the private Spanish haplotype involved in a reticulation is highlighted by an arrow. Haplotypes h1 and h2, mentioned in the text, are indicated. Networks for the other two PHAXs can be found in Supplementary Figure S3.

To better understand the phylogenetic relationships among haplotypes, and to provide insight into the reliability of the chosen PHAXs as non-recombining segments, we generated a median-joining network^36^ for each PHAX (Figure 4, Supplementary Figure S3). Only one reticulation was observed for PHAX 5574 (Figure 4) involving one nucleotide in a private Spanish haplotype, which could be compatible with either a recurrent mutation or a recombination event. This network also displays a pronounced ‘star-like' structure (Figure 4), in which two major haplotypes show high frequencies with several low-frequency haplotypes linked to them by one or two mutational steps; this seems compatible with a demographic expansion. One of the major haplotypes is h2 (in blue in Figure 3b), whereas the other haplotype shared across all populations is h1 (in green in Figure 3b). The majority of private YRI haplotypes lie outside the star-like part of the network. Two reticulations exist in the PHAX 3115 network (Supplementary Figure S3a). These both involve unique YRI haplotypes that interrupt the PHAX, whereas the segment still remains as an unbroken LD block in non-African populations. The star-like structure is less pronounced for PHAX 3115 compared with the other two PHAXs (Figure 4; Supplementary Figure S3b), but most branches are one or two mutational steps in length, with one branch carrying four mutation events. PHAX 8913 shows no reticulations in its network, suggesting that this region remains an LD block in the larger data set, lacking either recombination events or recurrent mutations (Supplementary Figure S3b). A high-frequency haplotype (h1) shared by all populations in the data set was found with several haplotypes at lower frequency linked to it only by single mutational events. This structure resembles the PHAX 5574 network and also seems compatible with a demographic expansion generating many haplotypes at low frequencies. Overall, the few reticulations found in the three PHAXs, mainly involving African haplotypes, confirm that such sequences remain useful haplotypic markers even when analysed in a larger European data set compared with the HapMap Phase II data used for their definition. Rare alleles found in non-African populations did not disrupt the LD blocks, and PHAXs still behave according to their original definition. Moreover, the general star-like structure found in all the three networks seems compatible with a demographic expansion that has generated many haplotypes at low frequencies branching from a few common haplotypes that are widely spread across populations.

### Demographic inferences

**Figure 5:**
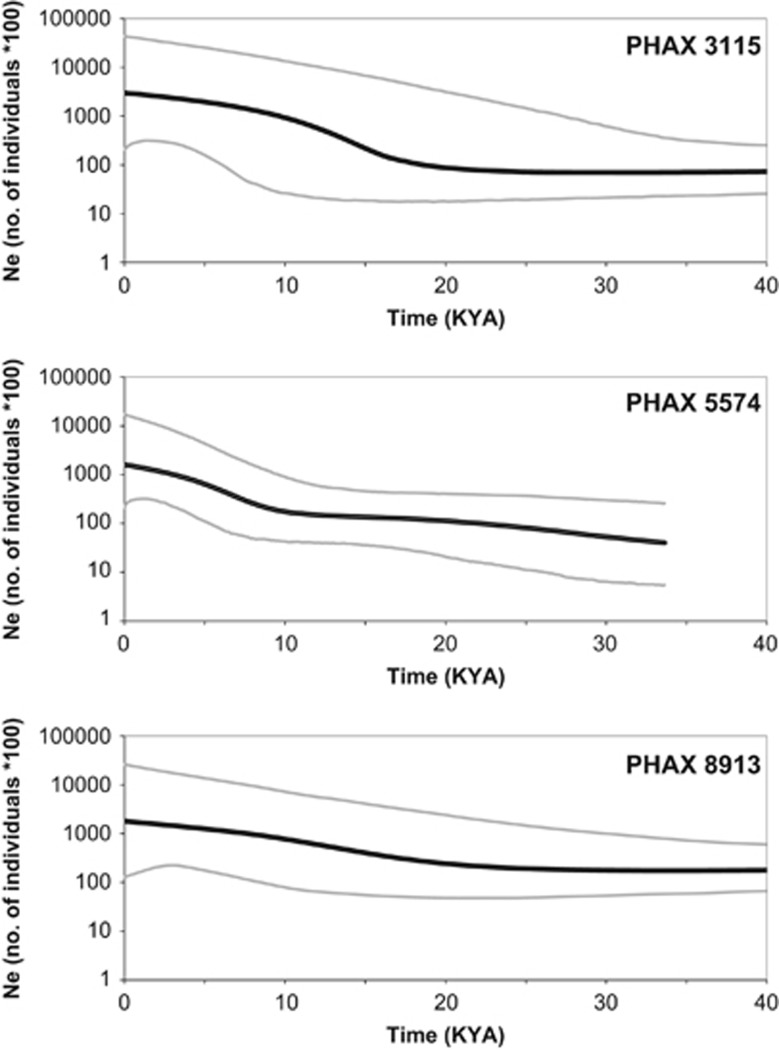
BSPs in European populations for the three PHAXs. Thick black lines indicate the median for effective population size (N_e_) and thinner grey lines show 95% higher posterior density intervals. For BSPs based on the YRI and Palestinian population samples, see Supplementary Figures S4 and S5.

BSPs were produced to analyse the demographic signal in Europe suggested by each PHAX. For this analysis, European populations (excluding YRI and Palestinians) were grouped together based on the low genetic distances suggested by the ϕst matrix and the absence of population structure indicated by the PCAs. The BSPs show an increase in effective population size of more than one order of magnitude (Figure 5), consistently across the three PHAXs. This increase starts around 20 KYA (thousand years ago) and becomes more pronounced around 18–15 KYA. PHAX 5574 shows a more constant pattern until 10 KYA, when the increase in effective population size becomes more marked. Overall, the patterns shown by the three PHAXs are compatible with an expansion in Europe starting around 20 KYA. By contrast, the YRI sample (Supplementary Figure S4) is characterised by flat BSPs that do not suggest any demographic change in effective population size. A similar pattern is seen for the Palestinian population (Supplementary Figure S5), with BSPs failing to show any strong demographic change. For both the YRI and Palestinian samples, all three PHAXs suggest comparable estimates of modern effective population size.

Time to most recent common ancestor (TMRCA) was calculated for specific haplotype clusters for each PHAX. Clusters were chosen based on the structures of networks – nodes of specific interest (such as the ancestral node) and prominent ‘star-like' structures (Supplementary Figure S6). Mean estimates with SD are reported in Table 2. All the ancestral nodes across the three PHAXs are dated between ~900 KYA and ~1.6 MYA. Cluster 1 in PHAX 3115 was dated ~46.8 KYA; this estimate is in agreement with two other clusters, cluster 2 (PHAX 5574) and cluster 1 (PHAX 8913), which have TMRCA estimates of ~44.85 KYA and ~44.82 KYA, respectively. Cluster 3 in PHAX 5574 is the youngest across the dated clusters showing TMRCA ~21.8 KYA, reflecting the very pronounced ‘star-like' structure in the network. Cluster 1 in PHAX 5574 divides almost all non-African samples from the African ones and has a TMRCA ~68.8 KYA.

## Discussion

Of the ‘odd couple' of the human sex chromosomes, the Y chromosome has received most attention in population genetics to date, because of its male specificity and the consequences for its mutation processes of its unusual state of constitutional haploidy. But the X chromosome is arguably just as interesting and strange, showing only one copy in males, female-biased mutation processes and the unique phenomenon of X inactivation.

The X chromosome also shows reduced crossover activity compared with autosomes, because the non-pseudoautosomal majority (98%) of the chromosome is recombinationally active in only one sex – females. We therefore expect it to contain relatively long segments that show limited historical recombination activity, and can be treated as simple haplotypes in evolutionary studies. The fact that males are haploid for the X chromosome means that studying such segments in males will provide reliably phased haplotypes, even when variants within these are very rare in the population. Here, we have investigated this idea by targeting three candidate X-chromosomal segments based on SNP data that indicate no evidence of historical recombination in the four HapMap Phase I populations. We identified such segments genome wide, and have designated them PHAXs.

High-coverage resequencing of the three PHAXs here, which cover 49 kb in total, reveals 297 SNPs in a sample of 240 males belonging to 12 populations, 11 of which are European or Middle Eastern. Almost 58% of SNPs are high-quality singletons suggesting that a resequencing approach is crucial for an accurate ascertainment of rare variants, which would have been lost with other techniques such as SNP arrays. Low-frequency sites provide vital information to finely assess and reconstruct recent demographic history. Network analysis shows that the absence of recombination generally persists in this independent sample in which variants are well ascertained. Two haplotypes showing evidence for recombination are found among the 20 males in the YRI population, whereas among 220 European and Middle Eastern chromosomes, one haplotype shows a single variant that requires recurrent mutation or recombination as an explanation. When additional available high-coverage sequence data from 13 samples sequenced by Complete Genomics are added to our data set, this pattern persists. HapMap Phase I data therefore seem a reliable source of information about non-recombining regions for evolutionary studies.

Unsurprisingly, the highest genetic diversity is found in the YRI population sample, consistent with genome-wide data on African *vs* non-African diversity.^23^ This can be seen in summary statistics (Table 3; Figure 2) and in the distribution of haplotypes in the networks (Figure 4; Supplementary Figure S3). In the non-African samples, diversity is lower, and the patterns of haplotypes in network analysis are more star-like, suggesting past population expansions.

The three PHAXs each show evidence of expansions across Europe starting around 20 KYA and pre-dating the Neolithic transition (beginning 10 KYA); in this, they are more consistent with the maternally inherited mtDNA, which shows expansion ~15–20 KYA^9, 10^ than with the male-specific Y chromosome, which shows expansions within the last 5 KY.^9, 10, 11^ In turn, this behaviour is compatible with the female bias of X-chromosomal inheritance, and underscores the importance of male-specific behaviours in the recent reshaping of the genetic landscape in Europe.

We also investigated the three PHAXs in two high-quality ancient male genomes: Loschbour (~8 KYA)^4^ and Ust'-Ishim (~45 KYA)^42^ from the Mesolithic and Palaeolithic, respectively (Supplementary Methods; Supplementary Table S7). Both match the commonest European haplotype for PHAX 3115. However, for the other two PHAXs Loschbour carries haplotypes compatible with rare haplotypes among our modern Europeans, whereas the Palaeolithic Ust'-Ishim carries haplotypes compatible with common modern European haplotypes. This finding is consistent with our conclusion of a Palaeolithic expansion in the histories of the studied PHAXs.

Each of the small number of PHAXs we have studied here is independently inherited, yet together they present a reasonably consistent picture of prehistory. The X chromosome contains a large number of additional PHAXs (180 additional examples identified across the whole X chromosome, excluding pseudoautosomal regions), and resequencing more would be desirable. In a much larger sample of PHAXs, we may expect to find a broader distribution of behaviours, including possible recent expansion haplotypes reflecting the Bronze Age migrations. Statistical approaches designed for multilocus data, incorporating sequence data from ancient DNA, would maximise the insights these loci can provide about the past, and in particular that of females.

## Figures and Tables

**Table 1:** Features of PHAXs analysed

*PHAX*	*Coordinates (hg19)*	*Length (bp)*	*No. of haplotypes defined by SNPs*^a^	*No. of HapMap (PII) SNPs (CEU)*	*No. of non-HapMap SNPs*^b^
3115	chrX: 39,519,963-39,524,945	4983	5	6	2
5574	chrX: 94,544,032-94,582,132	38 101	7	10	54
8913	chrX: 141,656,627-141,662,612	5986	5	5	44

a HapMap phase I/II populations (CEU, CHB, JPT and YRI).

b From dbSNP 152, insertion/deletions not included.

**Table 2:** TMRCA estimates

	*TMRCA/YBP*	*SD/years*
*PHAX 3115*		
Ancestral node	914 855	401 391
Cluster 1	46 850	14 055
*PHAX 5574*		
Ancestral node	1 194 432	212 395
Cluster 1	68 801	28 059
Cluster 2	44 847	14 500
Cluster 3	21 779	5731
*PHAX 8913*		
Ancestral node	1 590 917	489 355
Cluster 1	44 819	18 261

Abbreviations: TMRCA, time to most recent common ancestor; YBP, years before present.

For cluster definition, see Supplementary Figure S6.

**Table 3:** Genetic diversity of the three PHAXs

	*PHAX 3115*	*PHAX 5574*	*PHAX 8913*
*Number of segregating sites*			
Europe	15	64	42
YRI	16	142	35
Total	33	214	50
*Number of singletons*			
Europe	6	38	7
YRI	8	97	3
Total	20	141	11
*Number of haplotypes*			
Europe	13	54	20
YRI	12	18	10
Total	29	78	30
*Average φst*			
Europe	0.017	0.010	0.018
YRI	0.05	0.243	0.477
Total	0.023	0.048	0.096
*Nucleotide diversity (*π)			
Europe	0.072±0.043	0.012±0.007	0.054±0.032
YRI	0.101±0.060	0.109±0.056	0.236±0.124
Total	0.076±0.045	0.022±0.012	0.100±0.054
*Tajima's D*			
Europe	−0.24	−**2.37**	−**1.86**
YRI	−0.97	−**1.73**	0.78
Total	−**1.52**	−**2.70**	−1.16
*Fu's Fs*			
Europe	−1.05	−**26.78**	−4.84
YRI	−**4.64**	−2.78	2.11
Total	−**13.80**	−**25.17**	−4.76

In bold, significant values (*P*-value<0.05). Total: all populations included; Europe: European populations only (Palestinians excluded).
