# Supplementary material for: Signatures of human European Palaeolithic expansion shown by resequencing of non-recombining X-chromosome segments
Source: Eur J Hum Genet. 2017 Jan 25;25(4):485–92. doi: 10.1038/ejhg.2016.207 (PMC5386427; doi:10.1038/ejhg.2016.207)
Supplement: Supplementary Information [file ejhg2016207x1.docx]

**Supplementary Material for the manuscript entitled:**

**“Signatures of human European Paleolithic expansion shown by resequencing of non-recombining X-chromosome segments”**

**Supplementary Methods**

***PHAX identification process***

PHAXs were defined using publicly available polymorphism data from the HapMap project[^1^](#_ENREF_1), release 21, for four population samples: 60 Utah Residents with Northern and Western European ancestry (CEU), 60 individuals with African ancestry (Yoruba in Ibadan, Nigeria; YRI) and 90 individuals with East Asian ancestry: Japanese in Tokyo, Japan (JPT) and Han Chinese in Beijing, China (CHB). Haplotypes inferred using PHASE[^2^](#_ENREF_2) and measures of linkage disequilibrium were downloaded from the HapMap web site, release 16c.

Historically non-recombining regions were identified on both the autosomes and X chromosome as non-overlapping series of at least three adjacent SNPs. Each pair of SNPs was required to have a |D'| value of 1 in each of the three following populations: CEU, YRI and JPT+CHB, and only three of the four possible 2-allele haplotypes was allowed to be observed in the entire sample set (including the ancestral haplotypes, whether or not these were themselves observed in the data). When several such regions overlapped, the series spanning the largest physical distance was retained and the shorter overlapping regions were discarded. This procedure was repeated until no regions remained.

Ancestral states of SNPs were derived from the UCSC snp126orthopantro2recmac2 table. For a given SNP, the ancestral allele was taken to be the allele found in the chimpanzee and/or macaque sequence, provided it was observed in humans. Otherwise, the ancestral allele was taken to be the major allele in the global human sample (CEU+YRI+JPT+CHB). Series of adjacent SNPs where the ancestral states were not deducible from the chimpanzee or the macaque genomes, or where ancestral states of several SNPs were derived from several different chromosomes in the chimpanzee or macaque suggested unreliable orthologous alignments, and these regions were filtered out. Haplotypes containing the ancestral state of SNP alleles were derived for each PHAX, using SNPs for which genotypes were available in all four populations (CEU, YRI and JPT+CHB).

Genetic maps based on data from the HapMap release 20, HapMap release 21 and Perlegen data [^3^](#_ENREF_3) were analysed. To allow comparison of these three maps (HapMap20_LDmap, HapMap21_LDhat, Perlegen_LDhat), each map was re-computed using a sliding window of 2 kb. A distribution of the recombination rate was obtained for each map. Sequence annotation used genes, mRNA and repeats from the 'knownGenes', 'allmrna', 'rpmsk' and 'trf' tables from the UCSC web site.

***Variant calling and filtering***

Initially all sites were called, and then SNPs were selected and filtered. The samtools mpileup v0.1.19[^4^](#_ENREF_4) multi-sample option was used for multi-sample variant calling with the following general parameters: minimum base quality 20, minimum mapping quality 50 and no INDEL calling. In total 419 raw SNPs were called from 240 males. All sites with coverage <10 × were discarded and heterozygous sites also excluded. Clusters of heterozygous sites could suggest potential structural variation, but this has not been investigated in this study. In addition, sites and samples with >5% of missing data were discarded, giving a final dataset of 297 variants in 239 samples (sample SP15 was excluded). One sample (NTH005) was also excluded because it was discovered to be a female. This led to a final high-quality dataset made up of 297 variants in 238 samples.

***Sequencing error and validation***

*In silico* validation was done using Complete Genomics whole-genome sequence data (for nine samples in common) and Illumina sequence capture data[^5^](#_ENREF_5) encompassing the regions sequenced here (220 samples). Comparison versus the Complete Genomics samples was made on ~427,000 genotypes suggesting a false-positive rate of 0.0005% and false-negative rate of zero. The comparison versus the Illumina dataset was performed on 220 samples (~3.65 million genotypes including all sites that were retained after filtering, and shared between the Illumina and Ion Torrent dataset) suggesting a false-positive rate of zero and a false-negative rate 0.00003%. These low rates confirm the quality of the Ion Torrent sequencing dataset.

***Estimation of mutation rate***

Published estimates of the mutation rate on the X chromosome are often derived from introns or genes [^6-9^](#_ENREF_6). In order to avoid sequence bias, a different estimation of the mutation rate was carried out using a published dataset [^10^](#_ENREF_10).

The dataset called “masked” which excludes the fast-evolving sites of the genome was used. The sequence divergence on the X chromosome (D_x_) between human and chimpanzee was calculated as 6.44 ± 0.56 kbp^-1^ based on 8,002,847 sites[^10^](#_ENREF_10). The speciation time (T) is related to sequence divergence (D) and mutation rate (μ) by the formula$T=\frac{D}{2\mu}$. Speciation time between human and chimpanzee (T_HC_A_) was estimated as between 5.5 and 7 MYA using autosomal data[^10^](#_ENREF_10). At this stage, the α factor (ratio of mutations that occur in the male compared to the female germ-line) must be included to calculate the mutation rate. Therefore considering

$$1) T_{HC\_A}=\frac{D_{A}}{2\mu}$$

where D_A_ represents the sequence divergence calculated from autosomal data between human and chimpanzee

$${2) R}_{X/A}=\frac{D_{X}}{D_{A}}$$

where D_X_ represents the sequence divergence calculated from X-chromosomal data between human and chimpanzee and R_X/A_ represents the ratio between sequence divergence calculated from the X chromosome and from the autosomes. Therefore, 1) adapted for the X chromosome becomes

$$3) R_{X/A}\times T_{HC\_A}= \frac{D_{x}}{2\mu}$$

In this way the mutation rate on the X chromosome depends only on the sequence divergence (either based on autosomal or X-chromosomal data) and the speciation time based on autosomal data.

So considering 2), 3) is equal to

$$4) \mu=\frac{D_{X}}{2T_{HC\_A}}\times\frac{1}{R_{X/A}}$$

but the ratio between X chromosome and autosomes (R_X/A_) [^11^](#_ENREF_11), is

$${5) R}_{X/A}= \frac{2}{3} \times\left( 2+\alpha\right)\times\frac{1}{(1+\alpha)}$$

so the mutation rate on the X chromosome can be calculated as

$$6) \mu=\frac{D_{X}}{2T_{HC\_A}}\times\frac{1}{\frac{2}{3} \times\left( 2+\alpha\right)\times\frac{1}{(1+\alpha)}}$$

At this stage, D_X_ can be approximated to 6.44 × 10^-3^ bp (ref. [^12^](#_ENREF_12)), and the upper and the lower value for the speciation time between human and chimpanzee can be used to calculate a distribution of the mutation rate with α = 4.3 (modified from ref. [^13^](#_ENREF_13)).

So from 6), the upper estimate of the mutation rate was calculated using T_HC_A_ = 5.5 MYA

$$7) \mu_{upper}=\frac{6.44 \times{10}^{-3} bp}{2 \times5.5 \times{10}^{6} years}\times\frac{1}{\frac{2}{3} \times\left( 2+4.3 \right)\times\frac{1}{(1+4.3)}}=7.38 \times{10}^{-10} per site per year$$

Then the lower estimate of the mutation rate was calculated using T_HC_A_ = 7 MYA

$$8) \mu_{lower}=\frac{6.44 \times{10}^{-3} bp}{2 \times7\times{10}^{6} years}\times\frac{1}{\frac{2}{3} \times\left( 2+4.3 \right)\times\frac{1}{(1+4.3)}}=5.81 \times{10}^{-10} per site per year$$

Considering the narrow interval defined by the upper and lower estimates of the mutation rate, the mean value was calculated (6.59 x 10^-10^ per site per year) and used for the demographic inferences.

***Descriptive analyses***

Principal component analysis (PCA) was performed for each PHAX using two datasets: “full dataset” including all populations (Figure S1) and “reduced dataset” where the Yoruba and the Palestinian populations were excluded for all three PHAXs and for PHAX 3115 only respectively (Figure S2). This approach allowed a better resolution of population distribution in the PCA. PCAs were performed on haplotype frequencies per population with the FactoMineR statistical package[^14^](#_ENREF_14).

Genetic distances between pairs of populations were measured using φst^[15](#_ENREF_15)^, which is analogous to Fst^[16](#_ENREF_16)^ but at a nucleotide sequence (haplotype) level, computed with Arlequin v3.5[^17^](#_ENREF_17). The pairwise distance matrix for each PHAX is shown in Table S5.

***Consideration of additional publicly available sequence data***

Additional comparable sequence data on 13 unrelated male samples from six populations were publicly available from the Complete Genomics whole-genome sequence dataset (<http://www.completegenomics.com/public-data/69-genomes/>, Complete Genomics assembly software: 2.0.0.26) [^18^](#_ENREF_18), and these were coanalysed with the data from the current study to increase the sample size in Africa and Asia. This approach aimed to compare the genetic diversity of the PHAXs in Europe with a representation of the genetic diversity of the same sequences in Africa and Asia, and to ask if addition of these diverse samples would increase the number of observed recombination events.

Samples included were: NA18558 (Han Chinese from Beijing, China, CHB), NA18940 (Japanese from Tokyo, Japan, JPT), NA19020 (Luhya from Webuye, Kenya, LWK), NA19025 (LWK), NA19026 (LWK), NA20509 (Toscani from Italy, TSI), NA20510 (TSI), NA20511 (TSI), NA20845 (Gujarati Indians from Houston, Texas, GIH), NA20846 (GIH), NA20850 (GIH), NA21732 (Maasai from Kinyawa, Kenya, MKK) and NA21737 (MKK).

For each sample, variant sites were retrieved and compared with the Ion Torrent data set including 297 variant sites. Fourteen new sites were added to the dataset and haplotypes were re-estimated. The combined dataset included 311 variant sites and 251 samples. Overall, high genetic diversity and population structure was observed within Africa, by adding only five samples from two different populations (LWK, MKK). These samples show private haplotypes that are not shared with the YRI. This pattern was found for all three PHAXs and is consistent with the known high diversity among African populations[^19^](#_ENREF_19)^,^[^20^](#_ENREF_20). Haplotype h1 of PHAX 8913 (Figure 3c) with high frequency in Europe is also observed in Asia (Figure S7c), but none of the additional African samples carries it. A specific Eurasian haplotype could be a possible explanation even though the small sample size in Africa does not exclude its presence there. Haplotype h2 of PHAX 3115 (Figure 3a) remains apparently European and Middle East specific (Figure S7a). None of the additional samples carries it, either in Asia or in Africa. Sample sizes in both continents are small but preliminary indications would suggest its unique presence in this geographic area. Haplotype h2 in PHAX 5574 (Figure 3b) appears mainly distributed in Europe and Middle East with no sample carrying it in Asia and Africa (Figure S7b). This pattern could be compatible with a specific haplotype restricted to this geographic area but sample size effects cannot be rejected. The addition of five samples from two different Kenyan populations (LWW and MKK) increased the genetic diversity in Africa by introducing new haplotypes. Overall, ten new haplotypes were discovered across the three PHAXs (three for PHAX 3115, four for PHAX 5574 and three for PHAX 8913). Among the ten newly identified haplotypes only one (in PHAX 8913) is shared between the MKK and LWK, suggesting high diversity even between geographically close African populations. These patterns were also confirmed by the median-joining networks including these additional samples (Figure S8). Overall, the finding of new private African haplotypes confirms the high African genetic diversity and the complex pattern population structure in this continent.

***TMRCA estimation***

TMRCA estimation was also performed using BEAST v1.8[^21^](#_ENREF_21)^,^[^22^](#_ENREF_22). MCMC samples were based on 200,000,000 and 400,000,000 generations, logging every 10,000 and 20,000 steps, with the first 20,000,000 and 40,000,000 generations discarded as burn-in respectively. Traces were then manually evaluated and inspected using Tracer v 1.6 (<http://beast.bio.ed.ac.uk/software/tracer/>. TMRCA was estimated considering the whole dataset on individual PHAXs, always using a HKY substitution model[^23^](#_ENREF_23). Both an exponential growth and constant population size coalescent tree priors were explored suggesting comparable estimates (Table S6). Overall, the TMRCA estimates obtained using BEAST pre-date the Neolithic and they are in agreement with the estimates based on the *rho* statistic[^24^](#_ENREF_24)^,^[^25^](#_ENREF_25). The 95% high posterior density intervals are wide but the time for a possible overlapping with the Neolithic transition is not included.

***Surveying PHAX haplotypes in ancient genome sequences***

Two high-quality ancient male genomes were investigated: Loschbour (~8 KYA)[^26^](#_ENREF_26) and Ust’-Ishim (~45 KYA)[^27^](#_ENREF_27) from the Mesolithic and Paleolithic respectively. Aligned BAM files were downloaded and the X chromosome was then extracted. Variant calling was performed with samtools v. 1.3.1[^4^](#_ENREF_4) selecting all sites with a minimum base quality 20 and minimum mapping quality 30. Filtering was performed with a customised perl script discarding all sites with a depth of coverage < 4 ×. We found no private variants in the two ancient samples compared to the modern dataset. Polymorphic sites were used to compare the haplotypes of the ancient samples with the modern European dataset. Additional details on haplotype matching between the ancient and modern samples and haplotype frequency for each PHAX are reported in Table S7.

**References**

1. The International HapMap Consortium: The International HapMap Project: *Nature*, 2003, 2003/12/20 edn, Vol 426, pp 789-796.

2. Stephens M, Smith NJ, Donnelly P: A new statistical method for haplotype reconstruction from population data. *Am J Hum Genet* 2001; **68:** 978-989.

3. Peacock E, Whiteley P: Perlegen sciences, inc. *Pharmacogenomics* 2005; **6:** 439-442.

4. Li H, Handsaker B, Wysoker A *et al*: The Sequence Alignment/Map format and SAMtools. *Bioinformatics* 2009; **25:** 2078-2079.

5. Batini C, Hallast P, Zadik D *et al*: Large-scale recent expansion of European patrilineages shown by population resequencing. *Nat Commun* 2015; **6:** 7152.

6. Harris EE, Hey J: X chromosome evidence for ancient human histories. *Proc Natl Acad Sci U S A* 1999; **96:** 3320-3324.

7. Harding RM, Fullerton SM, Griffiths RC *et al*: Archaic African and Asian lineages in the genetic ancestry of modern humans. *Am J Hum Genet* 1997; **60:** 772-789.

8. Zietkiewicz E, Yotova V, Jarnik M *et al*: Genetic structure of the ancestral population of modern humans. *J Mol Evol* 1998; **47:** 146-155.

9. Kaessmann H, Heissig F, von Haeseler A, Paabo S: DNA sequence variation in a non-coding region of low recombination on the human X chromosome. *Nat Genet* 1999; **22:** 78-81.

10. Scally A, Dutheil JY, Hillier LW *et al*: Insights into hominid evolution from the gorilla genome sequence. *Nature* 2012; **483:** 169-175.

11. Miyata T, Hayashida H, Kuma K, Mitsuyasu K, Yasunaga T: Male-driven molecular evolution: a model and nucleotide sequence analysis. *Cold Spring Harb Symp Quant Biol* 1987; **52:** 863-867.

12. Scally A, Durbin R: Revising the human mutation rate: implications for understanding human evolution. *Nat Rev Genet* 2012; **13:** 745-753.

13. Rahbari R, Wuster A, Lindsay SJ *et al*: Timing, rates and spectra of human germline mutation. *Nat Genet* 2016; **48:** 126-133.

14. Le S, Josse J, Husson F: FactoMineR: An R Package for Multivariate Analysis. *Journal of Statistical Software* 2008; **25**.

15. Excoffier L, Smouse PE, Quattro JM: Analysis of molecular variance inferred from metric distances among DNA haplotypes: application to human mitochondrial DNA restriction data. *Genetics* 1992; **131:** 479-491.

16. Wright S: Genetical structure of populations. *Nature* 1950; **166:** 247-249.

17. Excoffier L, Lischer HE: Arlequin suite ver 3.5: a new series of programs to perform population genetics analyses under Linux and Windows. *Mol Ecol Resour* 2010; **10:** 564-567.

18. Drmanac R, Sparks AB, Callow MJ *et al*: Human genome sequencing using unchained base reads on self-assembling DNA nanoarrays. *Science* 2010; **327:** 78-81.

19. 1000 Genomes Project Consortium, Durbin RM, Abecasis GR *et al*: A map of human genome variation from population-scale sequencing. *Nature* 2010; **467:** 1061-1073.

20. Yu N, Chen FC, Ota S *et al*: Larger genetic differences within africans than between Africans and Eurasians. *Genetics* 2002; **161:** 269-274.

21. Drummond AJ, Rambaut A: BEAST: Bayesian evolutionary analysis by sampling trees. *BMC Evol Biol* 2007; **7:** 214.

22. Drummond AJ, Rambaut A, Shapiro B, Pybus OG: Bayesian coalescent inference of past population dynamics from molecular sequences. *Mol Biol Evol* 2005; **22:** 1185-1192.

23. Hasegawa M, Kishino H, Yano T: Dating of the human-ape splitting by a molecular clock of mitochondrial DNA. *J Mol Evol* 1985; **22:** 160-174.

24. Forster P, Harding R, Torroni A, Bandelt HJ: Origin and evolution of Native American mtDNA variation: a reappraisal. *Am J Hum Genet* 1996; **59:** 935-945.

25. Saillard J, Forster P, Lynnerup N, Bandelt HJ, Norby S: mtDNA variation among Greenland Eskimos: the edge of the Beringian expansion. *Am J Hum Genet* 2000; **67:** 718-726.

26. Lazaridis I, Patterson N, Mittnik A *et al*: Ancient human genomes suggest three ancestral populations for present-day Europeans. *Nature* 2014; **513:** 409-413.

27. Fu Q, Li H, Moorjani P *et al*: Genome sequence of a 45,000-year-old modern human from western Siberia. *Nature* 2014; **514:** 445-449.
