# Supplementary figures and images for: Signatures of human European Palaeolithic expansion shown by resequencing of non-recombining X-chromosome segments
Source: Eur J Hum Genet. 2017 Jan 25;25(4):485–92. doi: 10.1038/ejhg.2016.207 (PMC5386427; doi:10.1038/ejhg.2016.207)

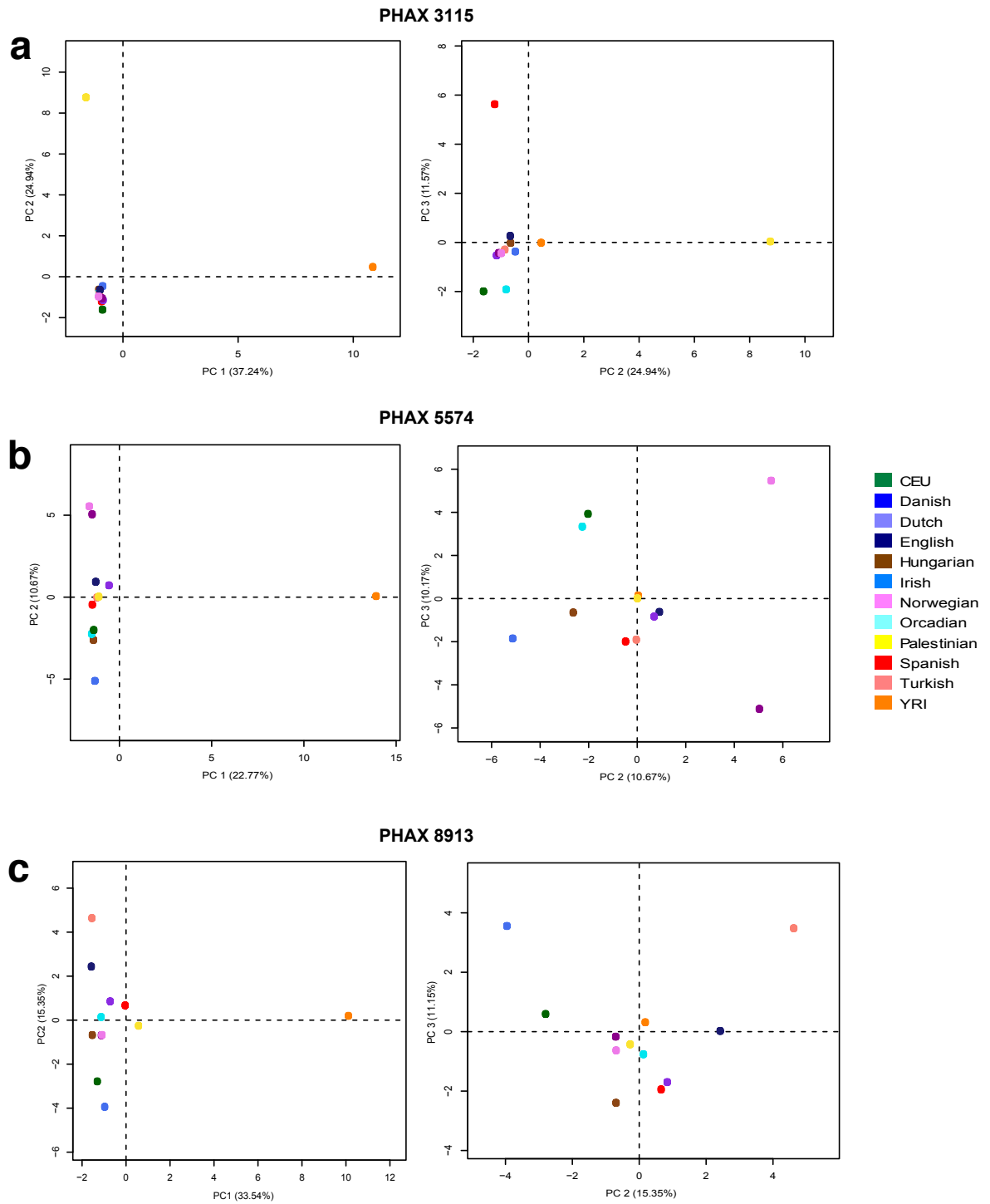

**Figure S1: PCA for all three PHAXs including all populations analysed.**

Supplement: Supplementary Information [file ejhg2016207x2.pdf]
