# Supplementary material for: Signatures of human European Palaeolithic expansion shown by resequencing of non-recombining X-chromosome segments
Source: Eur J Hum Genet. 2017 Jan 25;25(4):485–92. doi: 10.1038/ejhg.2016.207 (PMC5386427; doi:10.1038/ejhg.2016.207)
Supplement: Supplementary Information [file ejhg2016207x3.pdf]

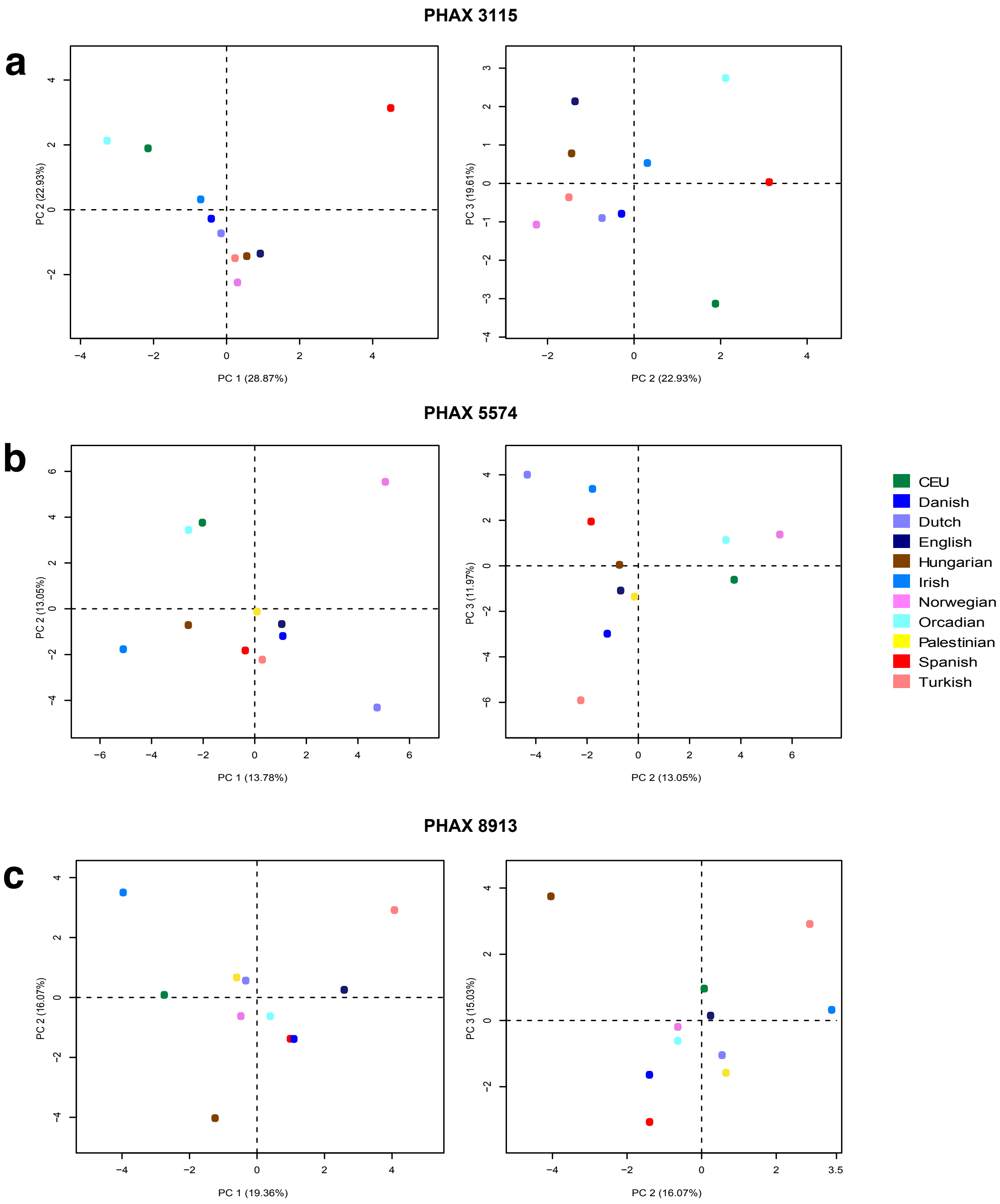

Figure S2: PCA for all three PHAXs omitting the YRI.
Palestinian samples were also excluded for PHAX 3115.
