# Supplementary material for: Signatures of human European Palaeolithic expansion shown by resequencing of non-recombining X-chromosome segments
Source: Eur J Hum Genet. 2017 Jan 25;25(4):485–92. doi: 10.1038/ejhg.2016.207 (PMC5386427; doi:10.1038/ejhg.2016.207)
Supplement: Supplementary Information [file ejhg2016207x4.pdf]

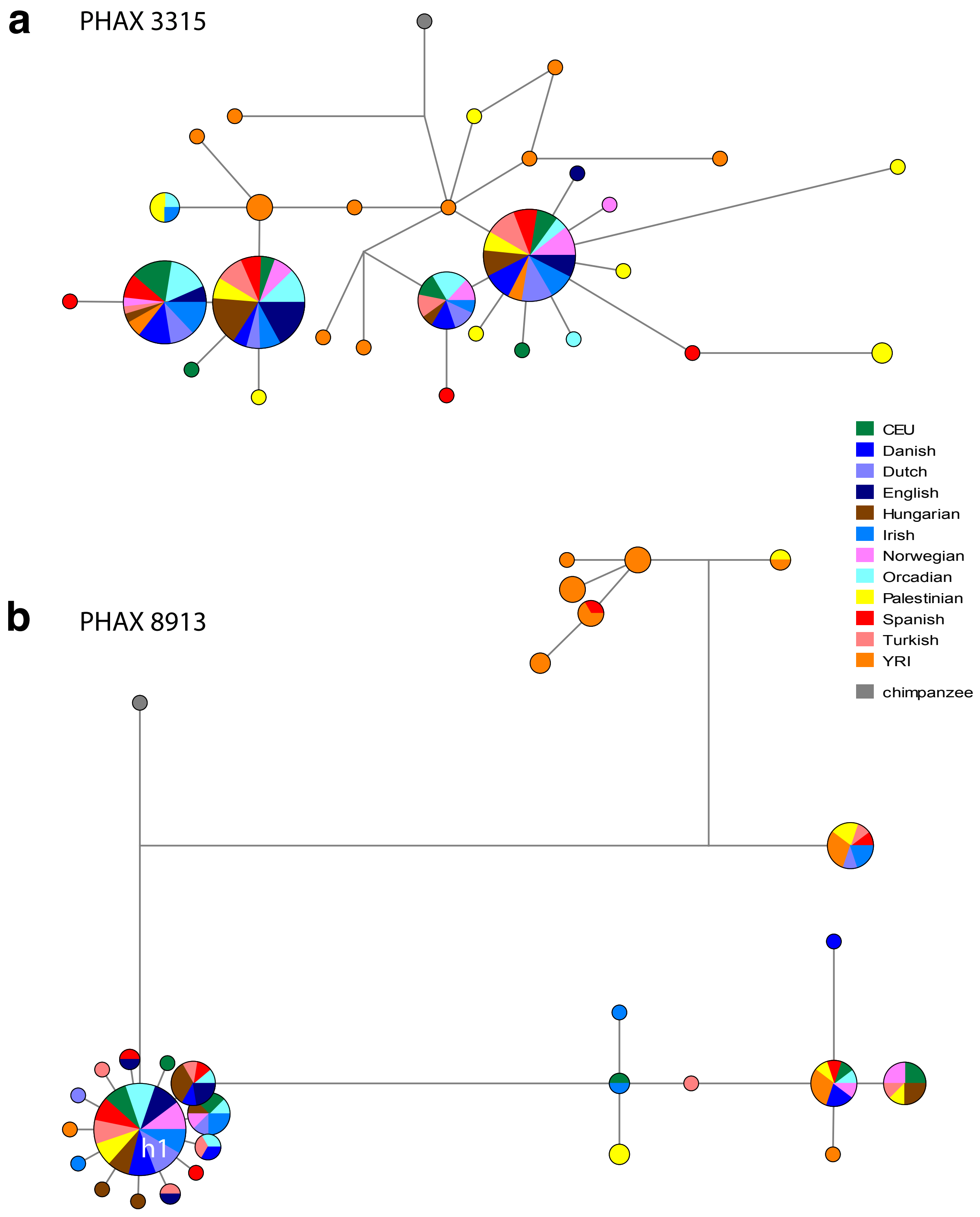

Figure S3: Median-joining networks for PHAXs 3115 and 8913.
Circles represent haplotypes with area proportional to frequency, and lines between them represent SNP mutational steps between haplotypes. Populations are indicated by colours as shown in the key to the right. Haplotype h1, mentioned in the text, is indicated in panel (b). Networks for PHAX 5574 can be found in Figure 4.
