# Supplementary material for: Signatures of human European Palaeolithic expansion shown by resequencing of non-recombining X-chromosome segments
Source: Eur J Hum Genet. 2017 Jan 25;25(4):485–92. doi: 10.1038/ejhg.2016.207 (PMC5386427; doi:10.1038/ejhg.2016.207)
Supplement: Supplementary Information [file ejhg2016207x7.pdf]

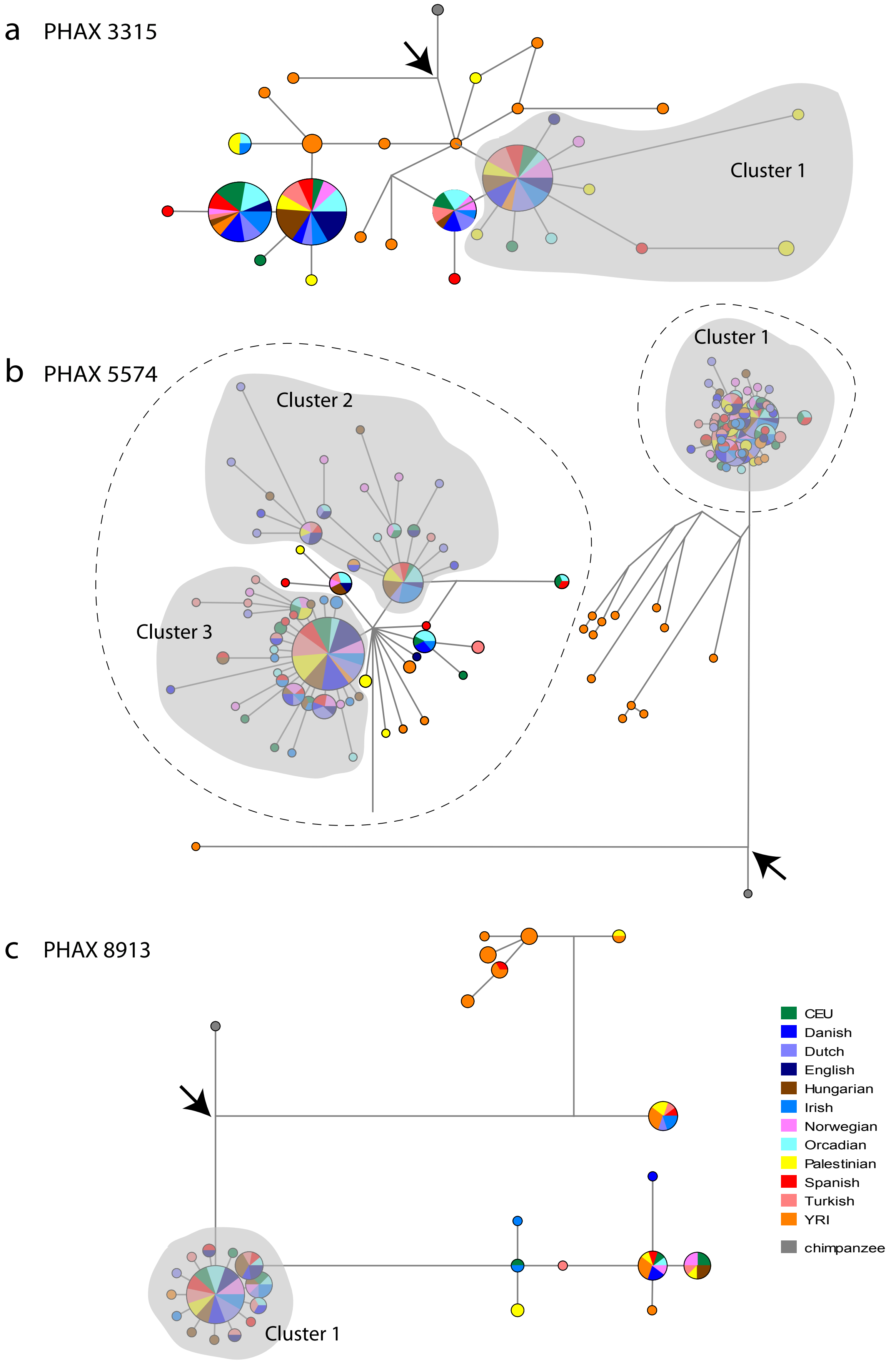

Figure S6: Median-joining networks showing clusters of haplotypes used in dating. Networks are shown as in Figures 4 and S3. Clusters used for dating (Table 3) are indicated by grey shading, and arrows indicate ancestral nodes.
