# Supplementary material for: Signatures of human European Palaeolithic expansion shown by resequencing of non-recombining X-chromosome segments
Source: Eur J Hum Genet. 2017 Jan 25;25(4):485–92. doi: 10.1038/ejhg.2016.207 (PMC5386427; doi:10.1038/ejhg.2016.207)
Supplement: Supplementary Information [file ejhg2016207x8.pdf]

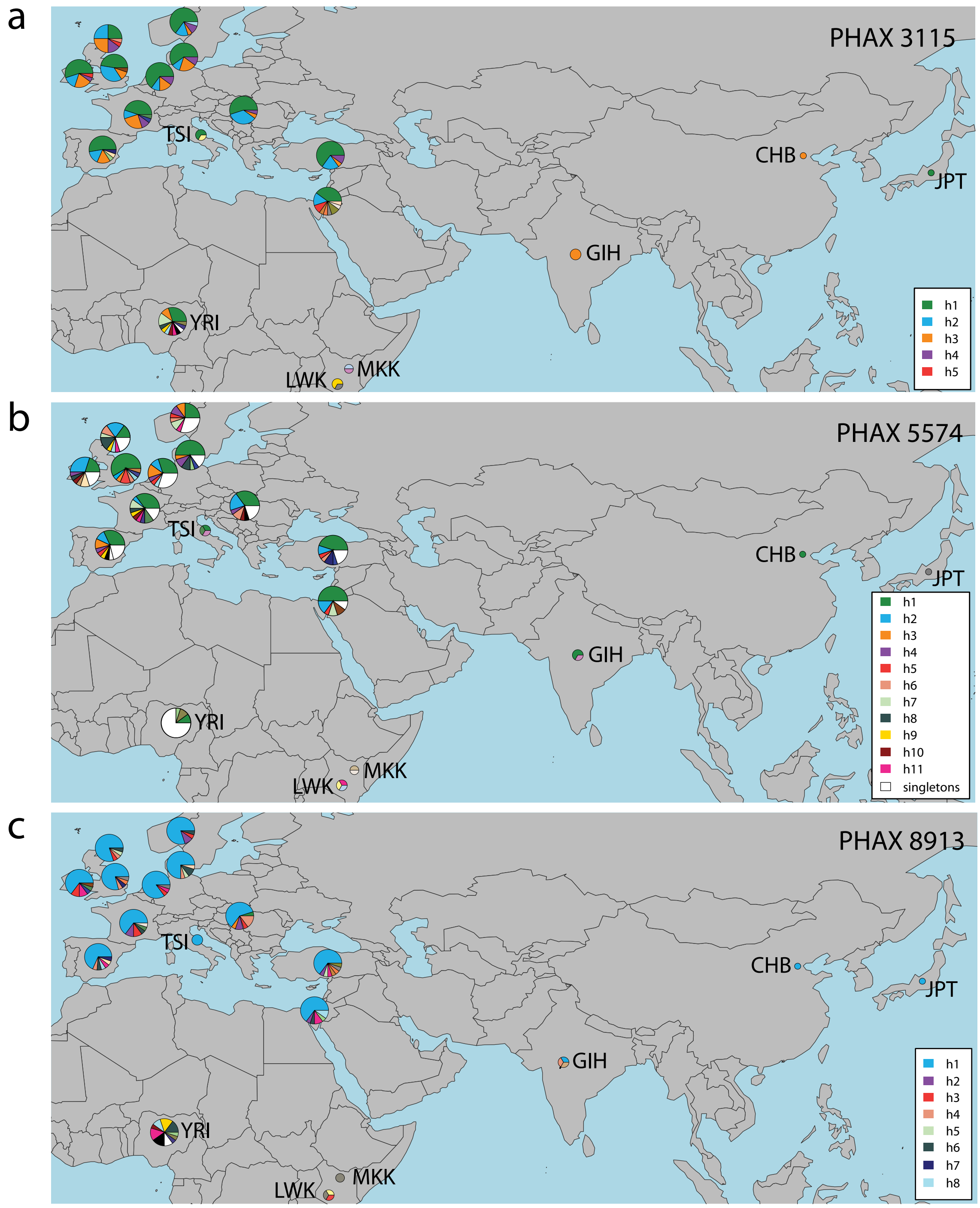

Figure S7: Population distributions of haplotypes, including the Complete Genomics data. Maps showing distributions of haplotypes for each PHAX, indicated by coloured sectors in pie charts. (a) PHAX 3115: the key indicates non-singleton European haplotypes (h1-5). (b) PHAX 5574: the key indicates haplotypes (h1-11) present in three or more European individuals; white sectors in pie charts correspond to singleton haplotypes in the European+YRI dataset. (c) PHAX 8913: the key indicates non-singleton European haplotypes (h1-8). Population abbreviations are as follows: YRI: Yoruba from Ibadan, Nigeria; CHB: Han Chinese from Beijing, China; JPT: Japanese from Tokyo, Japan; LWK: Luhya from Webuye, Kenya; TSI: Toscani from Italy; GIH: Gujarati Indians from Houston, Texas; MKK: Maasai from Kinyawa, Kenya.
