# Supplementary material for: Signatures of human European Palaeolithic expansion shown by resequencing of non-recombining X-chromosome segments
Source: Eur J Hum Genet. 2017 Jan 25;25(4):485–92. doi: 10.1038/ejhg.2016.207 (PMC5386427; doi:10.1038/ejhg.2016.207)
Supplement: Supplementary Information [file ejhg2016207x9.pdf]

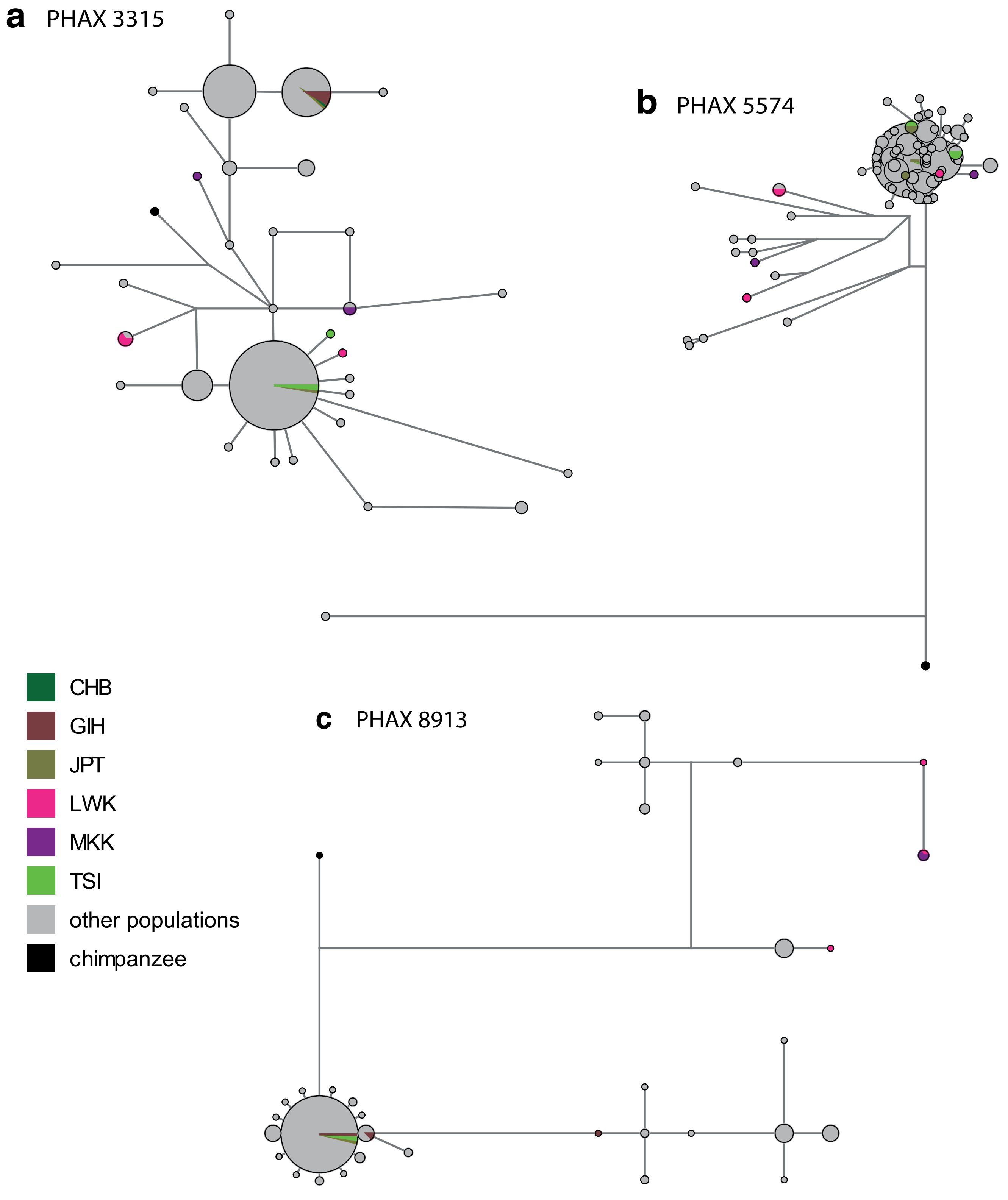

Figure S8: Median-joining networks including additional samples from Complete Genomics dataset.
Networks are shown as in Figures 4 and S3. Additional samples from the Complete Genomics dataset are highlighted by colours for the six populations as shown in the key, and the chimpanzee haplotype is also indicated.
